# Ensuring Safe Drinking Water: Physicochemical Analysis of Water Sources in Malle Woreda, South Omo Zone, Ethiopia

**DOI:** 10.1155/ianc/6911456

**Published:** 2025-10-30

**Authors:** Manayesh Adimase Bogale, Woldesenbet Bafe Dilebo, Tsirsit Tereke Kidane, Kero Assefa Ago, Meselu Eskezia Ayalew, Mihretu Bafe Dilebo

**Affiliations:** ^1^Department of Chemistry, College of Natural and Computational Science, Jinka University, Jinka, Southern Ethiopia, Ethiopia; ^2^Department of Chemistry, Oda Bultum University, Chiro, Ethiopia

**Keywords:** bore-well water, drinking water quality, physicochemical parameters

## Abstract

Water is essential for human life, yet contaminated drinking water poses significant health risks, leading to various waterborne diseases. The quality of drinking water is primarily determined by its physicochemical and biological characteristics, making regular monitoring crucial. However, no prior studies have assessed the physicochemical properties of drinking water in Malle Woreda, South Omo Zone, southern Ethiopia. This study aims to evaluate the levels of selected physicochemical parameters, including pH, temperature, free chlorine, combined chlorine, nitrate (NO_3_^−^), nitrite (NO_2_^−^), ammonia (NH_3_), turbidity, electrical conductivity (EC), and fluoride (F^−^), in drinking water sources within the Malle district. Three water samples were randomly collected from three different kebeles: Gento, Kalendo, and Asheker. The results indicate that the measured values for temperature (25.0°C–27.23°C), pH (7.33–8.81), EC (102.4–124.1 µS/cm), turbidity (< 5 NTU), NH_3_ (0–0.1 mg/L), NO_3_^−^ (1.0–1.1 mg/L), NO_2_^−^ (0.1–1.0 mg/L), F^−^ (0.6–1.5 mg/L), free chlorine (0.1 mg/L), and combined chlorine (0–0.1 mg/L) generally meet the standards set by the World Health Organization (WHO) and the Ethiopian Standards Agency (ESA). Overall, the findings suggest that the protected spring water in Malle Woreda is suitable for drinking purposes. Compared to WHO and ESA guidelines, as well as studies from other regions, the drinking water in this area exhibits good physicochemical properties. Regular monitoring and management of water sources remain essential to ensure long-term water safety. Therefore, this study serves as a stepping stone for further investigations into additional water quality parameters.

## 1. Introduction

Access to safe drinking water is a fundamental human right and a critical component of public health [1]. Groundwater, in particular, plays a vital role in human life, with its quality influenced by factors such as atmospheric precipitation, inland surface water, subsurface geochemical processes, and the geology of a given area [2, 3]. Changes in recharge water composition, hydrological dynamics, and human activities further contribute to variations in groundwater quality over time [4]. In both rural and urban areas, groundwater remains the primary source of drinking water. However, in recent decades, rapid industrialization, urbanization, and agricultural expansion have led to significant groundwater contamination [5]. Pollutants, including heavy metals, nitrates, fluorides, and chlorides, often enter water supplies due to inadequate waste treatment and disposal, posing serious health risks [6]. Even in the absence of anthropogenic pollution, natural sources can introduce hazardous chemicals and metals into groundwater. As a result, waterborne diseases have become a widespread public health concern, particularly in developing regions where water quality monitoring is often insufficient [7].

The deterioration of groundwater quality is exacerbated by factors such as excessive use of chemical fertilizers, proximity of boreholes to septic tanks and waste disposal sites, poorly constructed wells, and reliance on shallow water sources [8]. Assessing the physicochemical characteristics of drinking water is crucial, as parameters such as pH, temperature, turbidity, conductivity, total dissolved solids, nitrates, ammonia, and fluoride directly impact human health [9]. Elevated levels of these contaminants can lead to a range of health issues, including gastrointestinal infections, neurological disorders, and organ damage [10]. Therefore, ensuring safe drinking water requires comprehensive water quality assessment and adherence to international guidelines, such as those established by the World Health Organization (WHO).

Regular monitoring of drinking water quality is essential to safeguard public health. Many international and national organizations, including WHO and the Ethiopian Standards Agency (ESA), have established permissible limits for various water quality parameters to prevent adverse health effects. While some pollutants can be detected through sensory observation such as changes in odor, taste, or color, others require chemical analysis to determine contamination levels accurately [11].

Despite the importance of water quality monitoring, limited research has been conducted on the physicochemical properties of drinking water in Malle Woreda, South Omo Zone, southern Ethiopia.

Malle Woreda, located in the South Omo Zone of Ethiopia, reflects the pressing water quality challenges commonly faced by rural communities. The area is predominantly agricultural, with a population of around 50,000 people, many of whom reside in remote locations with limited access to improved water sources. Most households rely on untreated spring water, leaving them vulnerable to contamination from agricultural runoff, domestic waste, and inadequate sanitation.

Previous studies by our research group in nearby South Aari Woreda (Aari Zone) revealed detectable contamination levels in local water sources, underscoring the need for comprehensive water quality assessments in similar regions. However, there has been no detailed investigation specifically focusing on the physicochemical properties of drinking water in these areas, including Malle Woreda. This gap highlights the need to assess water safety and suitability for human consumption more rigorously [12].

Malle Woreda's growing population, dependence on natural water sources, and minimal water treatment infrastructure create an urgent demand for scientific evaluation. Additionally, the area's susceptibility to climatic variability may further intensify water scarcity and quality issues. Understanding the physicochemical characteristics of local water sources is therefore crucial for identifying potential health risks and developing effective water management strategies. This study aims to conduct a comprehensive analysis of key physicochemical parameters including pH, temperature, turbidity, electrical conductivity (EC), free chlorine, total chlorine, nitrate (NO_3_^−^), nitrite (NO_2_^−^), ammonia, and fluoride (F^−^) in drinking water sources across Malle Woreda. The findings will be compared with national and international drinking water standards to determine their safety for human consumption. By providing critical insights into water quality, this research offers a valuable evidence base to guide local authorities and community stakeholders in improving access to safe drinking water. It also lays the groundwork for future studies and supports broader efforts toward sustainable water resource management in Ethiopia.

## 2. Methodology

### 2.1. Description of the Study Area

**Figure 1:**
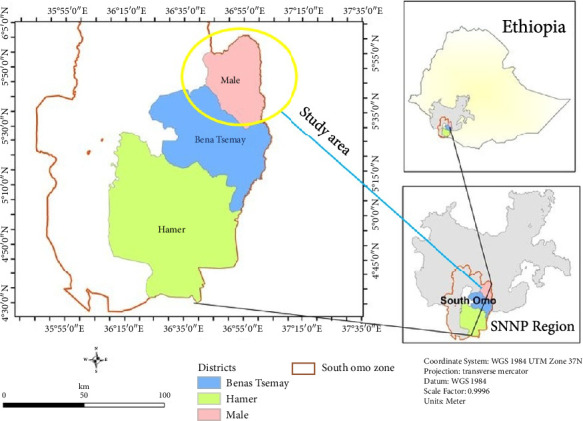
Location of the study area, shown in yellow on the map [13].

In this map, Malle Woreda is depicted in pink, situated in the southern part of the South Omo Zone. It shares borders with Bena Tsemay to the south and Hamer to the west. This region is part of the Southern Ethiopia Regional State, previously known as the Southern Nations, Nationalities, and Peoples' Region (SNNPR). This woreda is one of the eight woredas located in South Omo Zone which covers an area of 1432 square kilometers. The altitude of the woreda ranges between 600 and 1500 m above sea level and is astronomical located between 5.08°N–6.01°N lattitudinally and 36.30°E–37.0°E longitudinally. It had comprised 40% mid-altitude ranging from 1000 to 1400 m a.s.l, whereas 60% is low lands ranging from 605 to 999 m a.s.l. The mean annual rainfall which ranges from 800 to 1200 mm, and the mean annual temperature lies between 18°C and 35°C. The agropastoral production which is the farming system has prevailed in the study area for the last 4 decades [13, 14]. The corresponding study areas are indicated on the study map in Figure 1.

### 2.2. Instruments, Apparatus, Reagents, and Standards

#### 2.2.1. Instrumentation and Apparatus

Polyethylene bottles, polyethylene bags, ice box, a mercury thermometer (0–100°C),(Wagtech, China), turbidity meter (Wagtech, China), pH-meter (Wagtech, China), portable conductivity meter (Wagtech, China), volumetric flasks (25, 50, 100, and 250) mL, volumetric pipettes (1, 5, 10, and 15) mL, burette (50 mL), measuring cylinders (10, 25, and 50 mL), Erlenmeyer flasks (150 mL), beakers (250 mL), conductivity meter, refrigerator, and mercury thermometer (°C) were used.

#### 2.2.2. Chemicals and Standard Solutions

The chemicals used in the analysis were of analytical grade. These included 35.4% HCl (Loba Chemical Ltd., Laboratory Reagent, India), Wagtech DPD1, buffer solutions, Wagtech ammonia, Wagtech Nitrocol, fluoride, HNO_3_, and alcohol.

### 2.3. Sampling Procedures

#### 2.3.1. Cleaning of Sampling Equipment

Sampling bottles were cleaned using a metal-free soap, followed by soaking in 10% (v/v) HNO_3_ for 24 h. They were then rinsed thoroughly with distilled water, air dried, and stored with caps on to prevent contamination. Before sample collection, bottles were rinsed with the sample water to avoid impurities.

#### 2.3.2. Water Sampling and Transportation

Representative drinking water samples were collected from three different locations using prewashed polyethylene bottles and the grab sampling technique. Each container was labeled on-site with an appropriate code. Samples were transported to the laboratory and stored at 4°C until analysis (APHA, 1999).

### 2.4. In Situ Analysis

#### 2.4.1. Temperature Measurement

Water temperature was measured in situ using a mercury thermometer (range: 0°C–100°C). Prior to measurement, the thermometer was calibrated against a standard reference following APHA (1999) guidelines. Each sample was allowed to equilibrate fully before readings were recorded.

#### 2.4.2. EC

EC was measured using a digital conductivity meter, following the manufacturer's instructions. Prior to each measurement, the probe was rinsed with distilled water to ensure accuracy. The probe was then immersed in the water sample and gently stirred to eliminate any trapped air bubbles before recording the readings.

#### 2.4.3. pH Measurement

pH was determined using a digital pH meter, calibrated with a standard pH 7.0 buffer solution in accordance with the manufacturer's instructions. Calibration was performed both before and after each set of measurements to ensure accuracy and reliability (APHA, 1999).

#### 2.4.4. Turbidity Measurement (Nephelometric Method)

Turbidity was measured using a turbidimeter based on the nephelometric method, which compares the intensity of light scattered by the sample with a standard reference suspension. A tungsten filament lamp served as the light source, while a photoelectric detector measured scattered light intensity. A clear, colorless glass tube was used for sample analysis (DR/2400 Spectrophotometer, 2004).

### 2.5. Laboratory Sample Analysis

#### 2.5.1. Classical Methods of Analysis

Parameters including NO_3_^−^ analysis, NO_2_^−^ analysis, fluoride analysis, free chlorine analysis, combined chlorine analysis, and ammonia analysis had been analyzed in Wagtheic method following the procedures given by a standard method (APHA, 0.1999) in SNNPR State Health Bureau Jinka Subregional Public Health Laboratory. All physicochemical measurements were performed on replicate samples (triplicates for each water source) to ensure accuracy, reliability, and reproducibility of the results.

## 3. Results and Discussion

Water samples were collected from three different sites in Malle Woreda S_1_ (Gento Kebele), S_2_ (Kalendo Kebele), and S_3_ (Asheker Kebele) all representing protected drinking spring water sources. These samples were analyzed to assess various physical and chemical parameters. In this study, only selected physicochemical analyses have been indicated for the presence of certain contaminants, but they do not provide information about that can pose serious health risks. Therefore, further study will be expected on this regard as this study hints out the gap of the study on the selected study area. Water may meet physicochemical standards yet still harbor harmful microorganisms. A summary of the measured values (*n* = 3) for the selected parameters is presented in the following sections. The results include the mean values and standard deviations of all measured physicochemical parameters in agreement with regulatory bodies' specific guidelines for physicochemical parameters.

Table 1 presents the mean values and standard deviations of the analyzed physicochemical parameters for the water samples collected from the three sites.

### 3.1. Common Physical Parameters Analyzed Through In Situ Methods

The results of the studied common physical parameters, including EC, turbidity, temperature, and pH that was analyzed on-site (in situ) measurements, are displayed in Table 2.

### 3.2. Comparison of the Results of This Study With the Reported Literature

We have attempted to compare the data of this study with the recent results reported by the other researchers.

### 3.3. EC

The EC values of water samples from different sites revealed notable variation. This measurements were conducted on in situ (on-site) measurements. Specifically, the distribution site (S_1_) exhibited the highest EC value at 124.1 µS/cm, followed by the spring water (S_2_) at 116.6 µS/cm and the reservoir (S_3_) at 102.4 µS/cm (Table 2). Thus, the observed EC values ranged between 102.4 and 124.1 µS/cm. A significant difference was noted between the conductivity of the spring water and that of the distribution site. Elevated EC generally indicates higher mineral content in the water. Variations in EC can be influenced by geomorphological context, aquifer depth, and the geological nature of surrounding soil formations [19].

**Figure 2:**
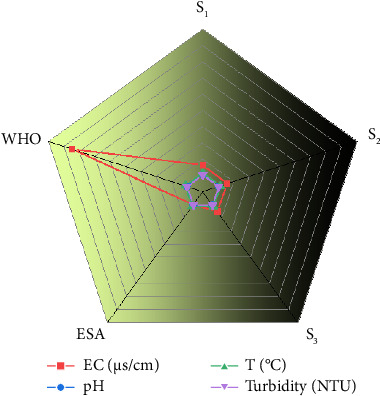
The mean values of in situ measured common physical parameters in the studied water samples.

In this study, the increasing trend in EC from the reservoir to the distribution site suggests a rise in mineral concentration, which may be due to the quality or sanitary status of the reservoir (S_1_) and/or contamination along the distribution pipeline to the outlet (S_3_). Nevertheless, all measured EC values were within the acceptable limits established by both the ESA and WHO guidelines (Figure 2), as well as consistent with the values reported in the recent literature (Table 3), indicating that the water is safe with respect to EC.

### 3.4. Hydrogen Ion Concentration (pH)

This also conducted on in situ analysis methods. The minimum and maximum pH values recorded were 7.33 and 8.18, observed in the spring water (S_3_) and reservoir water sample (S_1_), respectively. All pH values of the tested spring water samples fall within the acceptable ESA and WHO range of 6.5–8.5, as shown in Figure 2, as well as consistent with the values reported in the recent literature (Table 3), indicating that the water is safe for human consumption in terms of acidity/alkalinity. The results show that the pH of the water samples tends toward the basic side of the scale (pH > 7). Typically, spring water sources with pH values below 6.5 can be attributed to acidic discharges, often from agricultural runoff or domestic waste, affecting water bodies [18].

The basic pH observed in this study can also be linked to the geological composition of the region, which includes carbonate-rich bedrock. This aligns with the findings from previous research, indicating that approximately 98% of spring water characteristics worldwide are influenced by the geological nature of aquifer formations and surrounding landscapes [19].

### 3.5. Temperature

In the present study, water temperature ranged from 25.0°C to 27.23°C. All measured values fall within the permissible limits set by the WHO guidelines (Figure 2) as well as consistent with the values reported in the recent literature (Table 3). The relatively high temperature of the spring water in this study area may be attributed to the altitudinal characteristics of the region. Temperature is a critical parameter influencing both the physicochemical properties and biological processes in water systems. Elevated temperatures tend to accelerate chemical reactions and reduce the solubility of gases, which can impact water quality and aquatic life [20, 21].

### 3.6. Turbidity

Turbidity is an optical property of water, representing the extent to which light is scattered by suspended particles. Common contributors to turbidity in spring water include clay, silt, organic matter, phytoplankton, and other microscopic organisms [22]. Elevated turbidity can affect both esthetic quality and treatment efficiency, making its assessment essential for water quality monitoring.

**Figure 3:**
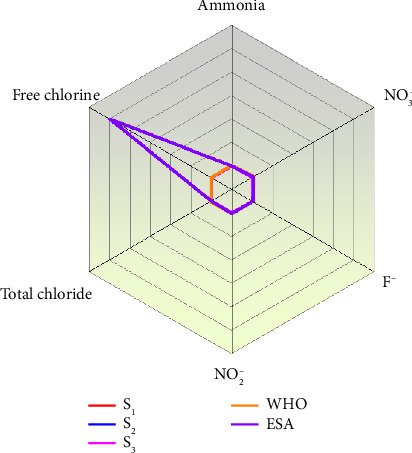
The mean values of laboratory measured common physical parameters in the studied water samples.

The turbidity of the water samples ranged from a minimum of 0.5 NTU in the spring water sample (S_1_) to a maximum of 4.9 NTU in the distribution site sample (S_3_). These values indicate relatively low levels of suspended particles across all sampling points. Notably, all turbidity measurements remained within the permissible limits set by the WHO (≤ 5 NTU) shown in Figure 3 and as well as consistent with the values reported in the recent literature (Table 3), suggesting that the water is visually clear. The slightly higher turbidity observed at the distribution site may reflect minor contamination or sedimentation during water transport, underscoring the need for regular monitoring to maintain water quality throughout the supply system [9].

### 3.7. Common Chemical Parameters Analyzed on Laboratory

The results of the studied common chemical parameters including ammonium, free chlorine, combined chlorine, fluoride, nitrite, and nitrate are displayed in Table 4.

#### 3.7.1. Fluoride Ion (F^−^)

The concentration of fluoride in the spring water samples was found to be within the maximum permissible limits set by both the WHO and the ESA for most of the sampled sites, including the spring water sample (S_1_), Reservoir (S_2_), and reservoir (S_3_). As presented in Table 4, fluoride levels in S_2_ and S_3_ were measured at approximately 1.5 mg/L, while S_1_ had a concentration of 0.6 mg/L. All values were within the WHO and ESA recommended limit of 1.5 mg/L for drinking water (Figure 3), as well as consistent with the values reported in the recent literature (Table 3), indicating that the water is safe for drinking purpose. Fluoride in natural water sources can originate from various industrial activities, such as iron and steel manufacturing, petroleum refining, and phosphate fertilizer production [23]. While adequate fluoride levels are beneficial for dental health, excessive concentrations can lead to dental and skeletal fluorosis. According to WHO, the optimal fluoride concentration in drinking water lies between 1.0 and 1.5 mg/L, with levels below 0.8 mg/L potentially leading to dental caries [24]. Therefore, it is crucial to maintain fluoride concentrations between 0.8 and 1.0 mg/L to ensure both health benefits and safety [25].

#### 3.7.2. Nitrite Ion (NO_2_^−^)

The mean nitrite concentration in the spring water samples was recorded in the range of 0.1–1 mg/L (Table 4), which is within the permissible limits set by the ESA and WHO guidelines (Figure 3), as well as consistent with the values reported in the recent literature (Table 3). This suggests that the water from these sources is safe for both domestic and livestock use. Typically, nitrites are absent in surface waters due to their unstable nature, as nitrogen compounds are more commonly found in the form of ammonia (NH_3_) or the more oxidized nitrate (NO_3_^−^). However, their presence in groundwater can occur under certain conditions, such as partial oxidation or contamination. The observed nitrite levels indicate that there is no immediate health risk associated with nitrite pollution in the sampled water sources.

#### 3.7.3. Free and Total Chlorine (Cl)

In the analyzed water samples, the concentration of free chlorine ranged from 1 to 1.1 mg/L, while combined chlorine ranged from 0 to 0.1 mg/L. All samples exhibited chloride concentrations within the permissible limits set by ESA and WHO, with the maximum allowable concentration being 250 mg/L in (Figure 3), as well as consistent with the values reported in the recent literature (Table 3). The lowest chloride level was detected at S_1_ (reservoir), as shown in Table 4, which may indicate the need for additional chlorination at that site to ensure disinfection. Chlorine levels in spring water were variable, which could be attributed to both natural presence and external sources such as weathering, leaching of sedimentary rocks, or seawater infiltration. According to Chadetrik and Arabinda, chloride concentrations between 250 and 500 mg/L can impart a salty taste to the water [6].

#### 3.7.4. Ammonia (NH_3_)

The mean concentration of ammonia in the spring water samples ranged from 0 to 0.1 mg/L, as shown in Table 4. These values are well within the permissible limits set by the ESA (1.5 mg/L) and WHO (0.5 mg/L), as shown in Figure 3, as well as consistent with the values reported in the recent literature (Table 3). This indicates the safe drinking water. Although relatively low, the presence of ammonia could be attributed to anthropogenic activities and fecal contamination, particularly from livestock breeding, the use of animal waste as fertilizer, wastewater runoff, and inadequate protection of the water sources [12]. These findings highlight the importance of improving source protection and sanitation practices to maintain water quality.

Overall, the findings of the current study indicate better overall water quality compared to the results reported by other researchers in various regions of Ethiopia. The pH values observed were more basic, yet remained within the acceptable range for potable drinking water, as shown in Table 4. The concentrations of nitrate (NO_3_^−^) and nitrite (NO_2_^−^) were higher in this study relative to previous reports, while EC and chlorine levels were found to be lower. Other parameters such as temperature, turbidity, fluoride, and ammonia were comparable to those reported by other researchers. These findings suggest that the water sources examined in this study are generally of good quality and suitable for domestic use, though continuous monitoring is recommended.

## 4. Conclusion

In this study, spring water samples from three different sites within Malle District, South Omo Zone, were analyzed to assess their physical and chemical properties. Key water quality parameters measured included pH, EC, temperature, turbidity, nitrate (NO_3_^−^), fluoride (F^−^), nitrite (NO_2_^−^), ammonia (NH_3_), free chlorine, and combined chlorine. The results indicate that, with the exception of elevated fluoride levels in Samples two and three, all parameters were within the acceptable limits set by the WHO and the ESA. Notably, Sample one had a more basic pH, while Sample two exhibited higher temperatures compared to the other sites. Overall, the findings suggest that the spring water in the three locations is generally suitable for drinking, though it may require moderate treatment, particularly in terms of chlorine disinfection, temperature adjustment, pH balancing, and fluoride reduction. The variation in water quality across sites is likely influenced by factors such as soil type, local climate, and anthropogenic activities. Compared to other studies conducted in different regions, the spring water in Malle Woreda demonstrates good physicochemical characteristics, making it a reliable source of drinking water. This research provides baseline data for ongoing monitoring and future assessments of drinking water quality in Malle Woreda and can inform local water resource management strategies.

### 4.1. Recommendation

The Wagtech method employed in this study proved effective for obtaining preliminary data on selected chemical parameters. However, for more accurate and comprehensive results, the use of advanced analytical instruments is recommended in future research. Based on the current findings, the spring water in Malle District requires additional treatment to ensure safety for consumption. Specifically, fluoride levels in samples two and three should be reduced, the pH in Sample one should be adjusted to fall within the optimal range, and the temperature in Sample two may require regulation. Additionally, chlorine should be added to Sample one to ensure safety. Furthermore, future studies should include the analysis of additional physicochemical parameters, such as major mineral ions (e.g., sodium, calcium, and magnesium), trace metals (e.g., arsenic, cadmium, and lead), radionuclides (e.g., uranium and radon), and other potentially carcinogenic substances. We suggest future research directions that include longitudinal studies on microbiological quality and its correlation with physicochemical parameters. Such assessments are essential for maintaining and enhancing the safety and quality of drinking water in the region.

The sampling design of this study is limited by the small number of water sources analyzed (three sites) and the absence of seasonal and broader spatial replication. Consequently, the results may not fully represent the overall water quality of Malle Woreda throughout the year. Future studies should include a larger number of sampling sites and incorporate multiseasonal sampling to capture spatial and temporal variations in water quality.

This study was conducted only during the rainy season, which may not fully capture seasonal variations in water quality. Since parameters such as rainfall, agricultural runoff, and environmental conditions can significantly influence water characteristics, the findings cannot be generalized to year-round drinking water safety. Future studies should include sampling across multiple seasons to provide a more comprehensive temporal assessment.

## Figures and Tables

**Table 1:** Physicochemical analysis results of water samples from Gento Kebele (Sample 1), Kalendo Kebele (Sample 2), and Asheker Kebele (Sample 3).

Parameters	S_1_	S_2_	S_3_	WHO	ESA
pH (in pH meter)	8.18	7.6	7.33	6.5–8.5	6.5–8.5
Conductivity in (μs)	124.06	116.63	102.4	185–1500	ND
Temperature in (^o^C)	25	27.23	26.7	25–28.1	25
Turbidity in (NTU)	< 5	< 5	< 5	< 5	5
Free chlorine in (mg/L)	0.1	0.1	0.1	0–0.1	250
Combined chlorine in (mg/L)	0	0.1	0.1	0–2.2	ND
Nitrite (NO_2_^−^) in (mg/L)	1	1	0.1	0–20	1.5
Ammonia in (mg/L)	0	0	0.1	0–1.5	ND
Fluoride in (mg/L)	0.6	1.5	1.5	0–1.5	1.5
Nitrate (NO_3_^−^) in (mg/L)	1	1	1.1	0–5	3

**Table 2:** The mean values of common physical parameters in the studied water samples.

No	Parameters	Sampling sites				
		S_1_	S_2_	S_3_	ESA	WHO
1	EC (μs/cm)	124.1	116.6	102.4	ND	1500
4	pH	8.18	7.6	7.33	6.5–8.5	6.5–8.5
5	T (°C)	25	27.23	26.7	25	25–28.1°C
6	Turbidity (NTU)	< 5	< 5	< 5	ND	5

Abbreviations: ESA, Ethiopia Standard Agency; NA, not assigned; ND, not detected; WHO, World Health Organization.

**Table 3:** Physicochemical data values of the current study and that of some recent works.

Parameters	Behailu et al. [15]	Reda [16]	Alemu et al. [17]	Shigut et al. [18]	Current study
pH	6.55–8.02	7.13–7.7	6.49–6.53	6.34–7.92	7.33–8.81
NH_3_	ND	ND	0.73–1.73	ND	0–0.1
E.C	0.396–1.77	150.76–158	1810.4 ± 73	ND	102.4–124.1
T	21.1–22.6	ND	27.4–28.4	ND	25–27.23
Turbidity	1.0–8.5	ND	ND	3.2–4.37	< 5
Free chlorine	14.79–193.3	260–271	15.77–16.83	ND	0.1-0.1
F^−^	0.088–0.78	2.048–4.42	ND	ND	0.6–1.5
NO_2_^−^	ND	BDL	ND	0.05–0.12	0.1–1
NO_3_^−^	ND	ND	ND	ND	1–1.1
Total chlorine	ND	ND	ND	ND	0–0.1

Abbreviations: ESA, Ethiopia Standard Agency; NA, not assigned; ND, not detected; WHO, World Health Organization.

**Table 4:** The mean values of common chemical parameters in the studied water samples in mg/L.

No	Parameters	Sampling sites				
		S_1_	S_2_	S_3_	WHO	ESA
1	Ammonia	0	0	0.1	0–1.5	NA
2	NO_3_^−^	1	1	1.1	0–5	3
3	F^−^	0.6	1.5	1.5	0–1.5	1.5
4	NO_2_^−^	1	1	0.1	0–2	1.5
5	Total chlorine	0	0.1	0.1	0–2.2	NA
6	Free chlorine	0.1	0.1	0.1	0–0.1	250

Abbreviations: ESA, Ethiopia standard Agency; NA, Not assigned; WHO, World health organization.

## Data Availability

The datasets generated and/or analyzed during the current study are original and authentic. The authors confirm that no data falsification or plagiarism is involved. In the event of any proven misconduct, the authors agree that the publication may be retracted.
